# Supplementary material for: Transient Receptor Potential Vanilloid 4-Dependent Microglial Function in Myelin Injury and Repair
Source: Int J Mol Sci. 2023 Dec 4;24(23):17097. doi: 10.3390/ijms242317097 (PMC10706888; doi:10.3390/ijms242317097)
Supplement: Supplementary file 1 [file ijms-24-17097-s001.zip › ijms-2643993-supplementary.pdf]

Supplemental Figure S1

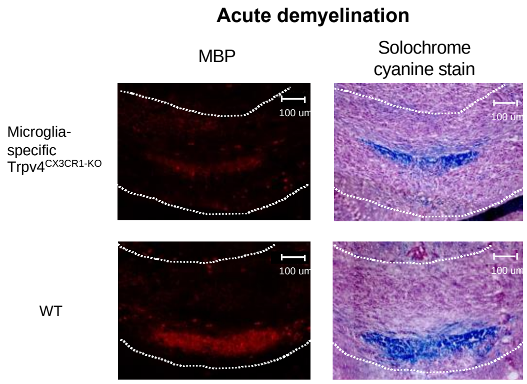

Supplemental Figure S1. Representative immunofluorescent and solochrome cyanine staining performed to ensure consistency of myelin staining. Serial sections taken from microglia-specific Trpv4$^{CX3CR1-}$KO and WT mice exposed to cuprizone-induced demyelination for six weeks were stained with fluorescent MBP and solochrome cyanine to ensure consistency of myelination staining across protocols. All images were taken of sections of the splenium of the corpus callosum. Fluorescent MBP stain (left column) and solochrome cyanine (right column, blue indicates myelination) both visualize myelination. Dotted white lines demarcate the borders of the splenium of the corpus callosum. Scale bars indicate 100 μm.

Supplemental Figure S2

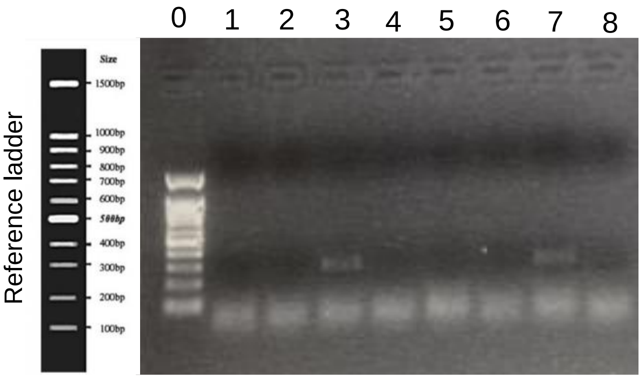

Supplementary Figure S2. PCR amplification indicating successful microglia-specific knockout of Trpv4 in the microglia of Cre-positive CX3CR1 Trpv4$^{f/f}$ mice (microglia-specific Trpv4$^{CX3CR1\text{-}KO}$ mice) treated with tamoxifen. Agarose gel of the PCR gene amplification products of DNA amplified with a primer specific for the recombined *Trpv4* gene (284 BP length). DNA was isolated from microglia and non-microglia CNS cells taken from Cre-positive and Cre-negative CX3CR1;Trpv4$^{f/f}$ mice after treatment with tamoxifen. Microglia were separated from non-microglia via flow cytometry. The experiment was run in duplicate with 4 mice total (2 Cre-negative mice and 2 Cre-positive mice). Well legend: 0 = ladder, 1 = Cre negative microglia, 2 = Cre negative non-microglia CNS cells, 3= Cre positive microglia, 4 = Cre positive non-microglia, 5 = Cre negative microglia, 6 = Cre positive non-microglia CNS cells, 7 = Cre positive microglia, 8 = Cre negative non-microglia CNS cells. Reference ladder included to left of the gel.
